# Neurolathyrism With Deep Vein Thrombosis and Bony Exostosis: Are They New Forms of Angiolathyrism and Osteolathyrism?

**DOI:** 10.7759/cureus.27720

**Published:** 2022-08-05

**Authors:** Pradeep Kumar, Arun Prasad, Giridhar M F, Sweta .

**Affiliations:** 1 Department of Pediatrics, All India Institute of Medical Sciences, Patna, IND

**Keywords:** deep vein thrombosis, exostosis, paraparesis, boaa, khesari dal, grass pea

## Abstract

Lathyriasis or lathyrism is a form of upper motor neuron disease caused by the dietary intake of grass pea (*Lathyrus sativus*). It is an irreversible crippling disease with poor outcomes. The possible pathogenesis is attributed to a toxin present in the legume, i.e., BOAA (beta-n-oxalyl amino L-alanine). Lathyrism can also be associated with vascular involvement resulting in angiolathyrism, which is mediated by a toxin β-aminopropionitrile, and bony involvement resulting in osteolathyrism characterized by bone growth impairment. A 12-year-old male child presented to us with chronic myalgia and a gradual decline in the power in the bilateral lower limbs, both in extension and flexion, followed by an inability to walk. On examination, he had spastic paraparesis with brisk deep tendon reflexes and positive Babinski sign with sustained bilateral ankle clonus suggestive of upper motor neuron lesion. Doppler studies of the bilateral lower limb suggested deep vein thrombosis of the right posterior tibial vein. His electrophysiological studies and neuroimaging were otherwise normal. We found deep vein thrombosis and bony exostosis, which have never been reported in the existing literature. This could be a new form of angiolathyrism and osteolathyrism we are reporting here. A review of dietary history revealed consumption of grass pea over the past few years daily, following which diagnosis of neurolathyrism was considered. A review of the literature does not suggest any specific treatment for this crippling disease and the treatment largely remains supportive. The child was provided vitamin C, gabapentin, and perampanel for neuromuscular pain, and low molecular weight heparin for deep vein thrombosis. Physiotherapy was initiated and surgical excision was planned by the orthopedic team for the exostotic lesion. The diagnosis of lathyrism should strongly be suspected if there is a history of consumption of grass pea. Public health education, improvement in the socio-economic condition, and strict prohibition of the sale and consumption of grass pea can root out the problem of lathyrism.

## Introduction

Neurolathyrism is a neglected tropical noninfectious disease affecting poor, drought-driven areas due to the neurotoxin BOAA (beta-n-oxalyl amino L-alanine) present in the legume grass pea (*Lathyrus sativus*). The disease is irreversible and crippling [1]. BOAA is a neurotoxin, an analog of L-glutamic acid [2]. L-BOAA acts as an excitatory neurotoxin at the AMPA (α-amino-3-hydroxy-5-methyl-4-isoxazole propionate) receptor [3]. Excitatory mechanisms are proposed to play a significant role in neurodegenerative disorders [4]. Secondary to toxin-mediated injury, specific involvement of Betz cells and corticospinal tracts are seen in neurolathyrism in humans causing spastic paraparesis [5]. The toxin mainly affects the nervous system but the skeletal and vascular systems may also get affected.

With the available evidence on lathyrism, it is still not known whether deep vein thrombosis and bony exostoses are the vascular and skeletal manifestations of lathyrism, respectively.

In this case report, we try to highlight the same, which might add a different dimension to the disease. In the regions of India like Bihar, poor people who cannot afford to eat common edible pulses are compelled to eat grass pea as a cheaper alternative and may acquire lathyrism. Health education about the toxic effects of eating grass pea, improved availability of common safe pulses, and prohibition of the sale of it are some of the important steps to eliminate this crippling disease.

There is no reported cure for this historical disease and its treatment is largely symptomatic, supportive, and rehabilitative.

## Case presentation

**Figure 1:**
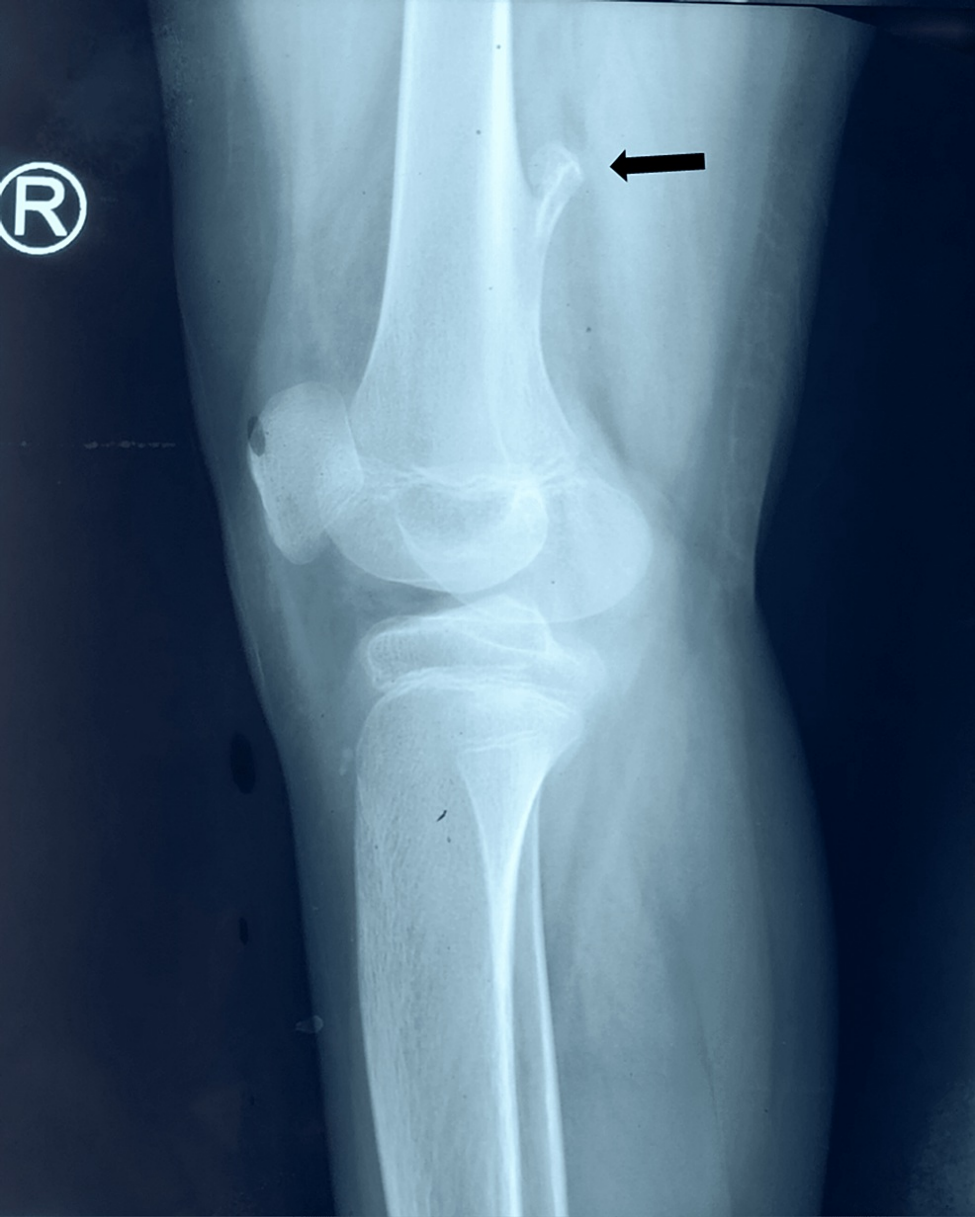
X-ray of the right knee (lateral view) showing bony exostosis in the right femur (black arrow)

Here, we report a 12-year-old male child from Bihar, India, who presented to us with pain in the bilateral lower limbs for six months and a gradual decline in the power of the bilateral lower limbs for four months. His birth history was not suggestive of perinatal asphyxia, and the child had normal development in all domains. On examination, he had spastic paraparesis with brisk deep tendon reflexes and positive Babinski sign with sustained bilateral ankle clonus suggestive of upper motor neuron lesion. Sensory system examination and cranial nerves were normal clinically. Bowel and bladder control was intact with normal urinary frequency. On palpation of the spine, tenderness was present in the lumbosacral region. Local examination of the right lower limb below the knee revealed erythema with rising temperature, with severe pain on movement of the limb, but peripheral pulses were normal. Deep palpation of the right knee showed some hard swelling posteriorly. There was no history of contact with an active case of tuberculosis. The child was non-ambulatory for over four months. Because of the above findings, Doppler studies of the bilateral lower limb were done, which were suggestive of deep vein thrombosis of the right posterior tibial vein, following which the child was started on low molecular weight heparin (LMWH) in a therapeutic dose. Nerve conduction velocity and electromyography studies were normal. X-ray of the lumbosacral spine, bilateral hip, and knee revealed exostosis arising from the right femur, and no features suggestive of ankylosing spondylitis were noted, with negative human leukocyte antigen B27 (HLA B27) (Figure 1).

Because of the presence of bony exostosis, we also searched for any presence of café-au-lait spot or any hormone excess state suggestive of McCune-Albright syndrome, but it was not found. Blood investigations did not show any significant abnormality except C-reactive protein (CRP) and erythrocyte sedimentation rate (ESR), which were mildly raised. The prothrombotic workup was also within normal limits (Table 1).

**Table 1:** Investigation reports CRP: C-reactive protein; ESR: erythrocyte sedimentation rate; HIV: human immunodeficiency virus; HBsAg: hepatitis B surface antigen; PT: prothrombin time; APTT: activated partial thromboplastin time; INR: international normalized ratio; HLA B27: human leukocyte antigen B27; NCV: nerve conduction velocity; EMG: electromyography; CEMRI: contrast-enhanced magnetic resonance imaging.

Investigation	Result	Reference value
Hemoglobin (g/dL)	10.7	12.0-15.0
Platelets (per cmm)	130×10^3^	150-450×10^3^
Leucocyte count (per cmm)	8620	4,000-11,000
Differential leucocyte count (%)		
Neutrophils	65.2	40-80
Lymphocytes	26.9	20-40
Monocytes	3.5	2-10
Eosinophils	9	1-6
Basophils	0	0-1
Peripheral blood smear	Predominantly microcytic hypochromic red cells admixed with few normocytes	
CRP (mg/dL)	8.69	0-5
ESR (mm in the first hour)	18	0-10
Serum bilirubin (total) (mg/dL)	0.41	0.3-1.2
Serum bilirubin (direct) (mg/dL)	0.09	<0.3
Aspartate aminotransferase (IU/L)	29.8	<31
Alanine aminotransferase (IU/L)	25.4	10-28
Alkaline phosphatase (IU/L)	152.2	100-290
Blood urea (mg/dL)	33.6	13-43
Serum creatinine (mg/dL)	0.57	0.7-1.3
Serum uric acid (mg/dL)	4.45	3.5-7.2
Serum sodium (mmol/L)	142.64	135-145
Serum potassium (mmol/L)	4.08	3.5-5
Serum calcium (mmol/L)	9.55	8.6-10
Serum phosphate (mmol/L)	4.29	2.7-4.5
HIV 1 and 2 serology	Negative	
HBsAg	Negative	
Hepatitis-C antibody	Negative	
Coagulation profile		
PT	14.72 seconds	<14 seconds
APTT	30.82 seconds	30-40 seconds
INR	1.13	0.8-1.1
HLA B27	3.91	<18
Rheumatoid factor and anti-cyclic citrullinated peptide (anti-CCP)	Negative	
NCV and EMG studies	Normal studies	
CEMRI of the brain with spine and bilateral hip	Normal studies	
USG Doppler of lower limbs	Deep vein thrombosis of right posterior tibial vein	

**Figure 2:**
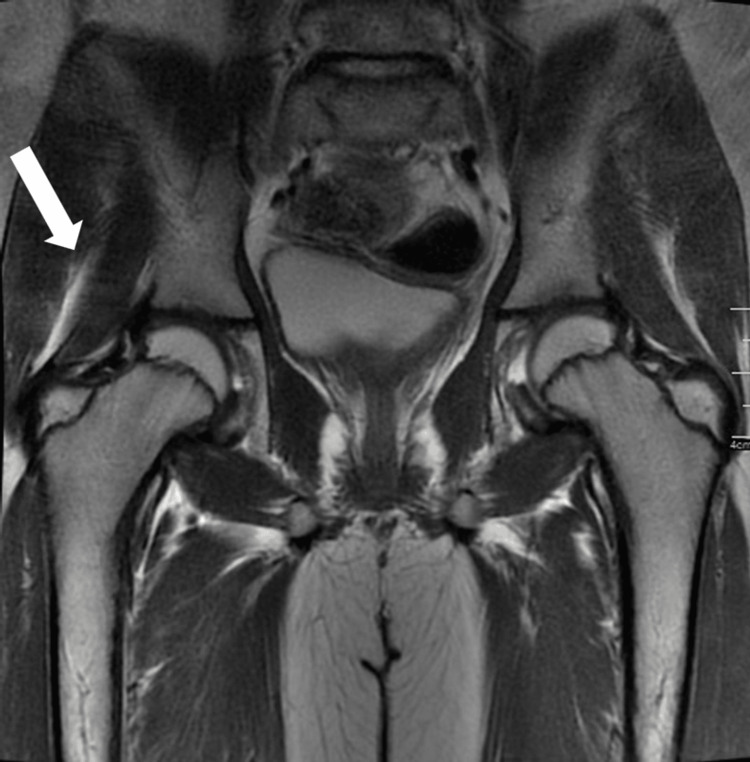
MRI of the pelvis showing normal soft tissues (white arrow)

Contrast-enhanced MRI of the brain with spine and hip was done, and studies were within normal limits. Spinal cord injury without radiographic abnormality (SCIWORA) was considered, but there was no history of antecedent trauma (Figure 2).

In view of the diagnostic dilemma, clinical history was thoroughly reviewed. On reviewing dietary history, it was found that the child was consuming grass pea over the past few years on daily basis in the form of soup, following which diagnosis of neurolathyrism was considered. We could not find any laboratory doing levels of BOAA in human blood in our area. Since neurolathyrism is a clinical diagnosis with a strong dietary history, the child was started on vitamin C supplementation. For severe pain, gabapentin and perampanel were started. Dietary counseling of the family was done, and the nature and prognosis of the disease were explained. Review Doppler studies showed resolution of the thrombus. Occupational therapy was initiated, and the child is currently under our follow-up. For exostotic lesions, we sought an orthopedic opinion, and surgical excision was planned by the orthopedics team. The patient is currently under their follow-up for surgical intervention.

## Discussion

Lathyriasis is a form of upper motor neuron disease caused by the large dietary intake of grass pea (*Lathyrus sativus*) [6]. The condition of neurolathyrism was investigated in India on a large scale by Buchanan (1904) [7]. The incidence of lathyriasis is highest in the state of Bihar when compared to other states of India [7].

The possible pathogenesis is attributed to a toxin present in the legume, i.e., BOAA [6]. BOAA is a neurotoxic analog of L-glutamic acid [2]. Glutamate is an excitatory neurotransmitter. There are three ligand-gated ionotropic glutamate channels, including NMDA (N-methyl-D-aspartate) and AMPA receptors [8]. L-BOAA acts as an excitatory neurotoxin at the AMPA receptor [3]. Excitatory mechanisms are proposed to play a significant role in neurodegenerative disorders [4]. Secondary to toxin-mediated injury, it was found that specific involvement of Betz cells and corticospinal tracts are seen in neurolathyrism in humans causing spastic paraparesis [5].

The child presented with severe spastic paraparesis with adductor spasm, exaggerated deep tendon reflexes, extensor plantar response, and ankle clonus. The onset may be subtle with pain in the lumbosacral region, which was seen in our patient. Other clinical features, including increased urinary frequency and urgency, can be seen. The severity of paraparesis is graded by the system that the patient uses for mobility, which includes no stick (mild), one stick (moderate), two sticks (severe), and crawler stage (very severe); our patient was in moderate (one stick) stage at the time of diagnosis [9,10]. Lathyrism can also cause vascular damage (angiolathyrism) such as dissecting aneurysm and damage to the bone growth (osteolathyrism) such as bowing of legs, kyphoscoliosis, or failure of fusion of the vertebral and iliac epiphyses [11-13]. Our patient had deep vein thrombosis and bony exostoses arising from the right femur, which could be a new presentation of angiolathyrism and osteolathyrism, respectively, which needs further research.

Our patient had a history of consumption of grass pea over a few years. Treatment of neurolathyrism is mainly symptomatic. In our case, we have started gabapentin and AMPA receptor antagonist perampanel. Tolperisone hydrochloride, a centrally acting muscle relaxant, had shown improvement in symptoms of varying degrees [14]. Vitamin C prophylaxis 500-1000 mg per day for a week was given [15]. In view of deep vein thrombosis, the child was started on LMWH, following which deep vein thrombosis got resolved, and ambulation improved a bit.

Neurolathyrism is an irreversible crippling disease with poor outcomes. Removal of grass pea prevents disease progression but disability attained is permanent. Therefore, public health education and socioeconomic changes can root out lathyrism.

## Conclusions

Lathyriasis is a diagnosis of exclusion and should strongly be suspected in a patient presenting with muscle weakness with or without bony exostosis with a history of consumption of grass pea, especially in resource-poor countries. Public health education, improvement in the socio-economic condition, and strict prohibition of the sale and consumption of grass pea are the only measures to get rid of the problem of lathyrism. Diet history carries significant weight, which is a key to alleviating the diagnostic dilemma in our case. Neurological abnormalities need not be always present in the imaging for making a diagnosis. Neurolathyrism, a neglected tropical noninfectious disease, has to be addressed on a higher scale.

## References

[1] Ngudi DD, Kuo YH, Van Montagu M, Lambein F (2012). Research on motor neuron diseases konzo and neurolathyrism: trends from 1990 to 2010. PLoS Negl Trop Dis.

[2] Kusama-Eguchi K (2019). Research in motor neuron diseases caused by natural substances: focus on pathological mechanisms of neurolathyrism. (Article in Japanese). Yakugaku Zasshi.

[3] Ravindranath V (2002). Neurolathyrism: mitochondrial dysfunction in excitotoxicity mediated by l-β-oxalyl aminoalanine. Neurochem Int.

[4] Tapia R, Medina-Ceja L, Peña F (1999). On the relationship between extracellular glutamate, hyperexcitation and neurodegeneration, in vivo. Neurochem Int.

[5] Ludolph AC, Spencer PS (1996). Toxic models of upper motor neuron disease. J Neurol Sci.

[6] Spencer PS, Roy DN, Ludolph A, Hugon J, Dwivedi MP, Schaumburg HH (1986). Lathyrism: evidence for role of the neuroexcitatory aminoacid BOAA. Lancet.

[7] Prasad LS (1973). Spastic paraplegia in lathyriasis. Paraplegia.

[8] Purves D, Augustine GJ, Fitzpatrick D (2001). Neuroscience. 2nd edition. White: Neuroscience Sinauer Associates Inc, Sunderland, Massachusetts.

[9] Schapira AHV, Byrne E (2007). Metabolic diseases. Neurology and Clinical Neuroscience.

[10] Dobbs MR (2009). Neurotoxic substances. Clinical Neurotoxicology E-Book: Syndromes, Substances, Environments.

[11] Sigler E, Shvidel L, Shtalrid M, Berrebi A (2007). Lathyrism, leg cramps, and thrombocytopenia: cascade of events starting in a concentration camp. Am J Med.

[12] Hosoda Y, Iri H (1966). Angiolathyrism. 1. A histological and histochemical study on successive changes of the lathyritic rat aorta. Acta Pathol Jpn.

[13] Haque A, Hossain M, Lambein F, Bell EA (1997). Evidence of osteolathyrism among patients suffering from neurolathyrism in Bangladesh. Nat Toxins.

[14] Haque A, Hossain M, Khan JK, Kuo YH, Lambein F, De Reuck J (1994). New findings and symptomatic treatment for neurolathyrism, a motor neuron disease occurring in north west Bangladesh. Paraplegia.

[15] Park K (2017). Nutrition and health. Park’s Textbook of Preventive Social Medicine.

